# Effects of Inositol Hexaphosphate and Myo-Inositol Administration in Breast Cancer Patients during Adjuvant Chemotherapy

**DOI:** 10.3390/jpm11080756

**Published:** 2021-07-30

**Authors:** Maria Ida Amabile, Alessandro De Luca, Domenico Tripodi, Elena D’Alberti, Rossella Melcarne, Giovanni Imbimbo, Orietta Picconi, Vito D’Andrea, Massimo Vergine, Salvatore Sorrenti, Alessio Molfino

**Affiliations:** 1 Department of Surgical Sciences, Sapienza University of Rome, 00161 Rome, Italy; mariaida.amabile@uniroma1.it (M.I.A.); dr.aless.deluca@gmail.com (A.D.L.); domenico.tripodi@uniroma1.it (D.T.); eledalberti@gmail.com (E.D.); rossella.melcarne@yahoo.it (R.M.); vito.dandrea@uniroma1.it (V.D.); massimo.vergine@uniroma1.it (M.V.); 2 Department of Translational and Precision Medicine, Sapienza University of Rome, 00185 Rome, Italy; giovanni.imbimbo@uniroma1.it (G.I.); alessio.molfino@uniroma1.it (A.M.); 3 National HIV/AIDS Research Center, Istituto Superiore di Sanità, 00161 Rome, Italy; oripic69@yahoo.it

**Keywords:** inositol hexaphosphate, myo-inositol, breast cancer, breast surgery, adjuvant chemotherapy, quality of life

## Abstract

Background: Treatment of breast cancer (BC) includes locoregional and systemic therapies depending on tumor and patient’s characteristics. Inositol hexaphosphate (IP6) is known as a strong antioxidant agent, able to improve local (i.e., breast region) side effects, functional status and quality-of-life. We investigated some potential beneficial effects, including hematological and local, of the combined therapy with oral myo-inositol administration and topical IP6 application in patients undergoing surgery for BC and eligible to adjuvant chemotherapy. Methods: We considered BC patients randomly assigned to the Inositol Group (oral myo-inositol + IP6 local application for the entire neoadjuvant treatment period) and to the Control Group (standard of care). The EORTC QLQ-BR23 and QLQ-C30 questionnaires were administered to both groups and blood parameters were assessed as per clinical routine practice at baseline (before starting adjuvant chemotherapy), T1 (after the first two doses of epirubicin-cyclophosphamide regimen), T2 (at the end of epirubicin-cyclophosphamide regimen), T3 (after the first six doses of paclitaxel regimen), and T4 (at the end of the paclitaxel treatment). Results: A total of 36 BC patients were considered, 18 in the Inositol Group and 18 in the Control Group. The Inositol Group showed a lower decrease in red blood cells, hemoglobin levels and white blood cells with respect to controls (*p* ≤ 0.02), as well as amelioration in scores related to breast and arm local symptoms (*p* ≤ 0.02), body image (*p* = 0.04) and quality-of-life related symptoms (*p* ≤ 0.04). Conclusions: In our cohort of BC patients, a combined treatment with oral myo-inositol + IP6 local application was able to improve local symptoms and quality-of-life related symptoms which represent clinically relevant aspects associated with patient’s prognosis.

## 1. Introduction

Breast cancer (BC) is the most common cancer diagnosed in women worldwide and it accounts for 522000 deaths globally [1]. In recent years, survival of patients affected by BC ameliorated due to several reasons, including the improved screening policies and more effective therapies [2,3].

Treatment of BC includes surgery, radiotherapy, chemotherapy, hormonal and immunological therapy depending on tumor and patient’s characteristics [4]. After locoregional treatment, BC patients can undergo systemic adjuvant treatments in order to improve the outcomes [4]. However, both locoregional treatment and systemic adjuvant treatments present several different side effects, including lymphedema, surgical site infections, as well as nausea, vomiting, body weight loss and anemia [5,6]. These adverse events may determine the interruption or the delay of the adjuvant therapies negatively affecting patient’s prognosis [7].

In this light, tackling adverse effects of anticancer treatments is crucial to ameliorate patient’s outcomes and quality of life [8,9,10,11].

Inositol hexaphosphate (IP6) is known as a strong antioxidant agent present in both plants and mammalian cells regulating signal transduction, cell differentiation and proliferation [12,13,14]. Several studies have investigated IP6 anticancer properties, trying to better understand the mechanisms of its action, in particular of myoinositol + IP6 supplementation [13,15,16]. The role of nutritional and metabolic intervention appears fundamental in the prevention and treatment of BC [17,18]. In fact, IP6 has been shown to reproducibly inhibit various cancers, including BC, through an apoptotic effect, as well as by synergizing with chemotherapy in inhibiting cancer growth [19,20].

The IP6 properties have been investigated in BC patients, in particular by a pilot study evaluating the effect of the oral administration of IP6 in BC patients documenting a significant amelioration in quality of life and functional status [21]. Moreover, Proietti et al. investigated the properties of IP6 topical formulation alone (directly applied on the surgical site) in BC patients observing an improvement in terms of local side effects and consequently improving functional status and quality of life [22]. Nevertheless, still very few organized clinical studies are available.

In addition, considering that it was hypothesized and shown that inositol potentiates the anticancer properties of IP6 [23,24], we aimed to assess the beneficial effects of the combined therapy with oral myo-inositol administration and topical IP6 application in patients undergone surgery for BC and eligible to adjuvant chemotherapy on hematological, systemic, and local side effects.

## 2. Materials and Methods

### 2.1. Patients

We conducted a prospective spontaneous, single-center, controlled study on patients from the Department of Surgical Sciences, Sapienza—University of Rome, Italy. After approval of the local Ethics Committee (Ethics Committee of Sapienza University of Rome, Rome, Italy, ref. n. 5050) and after obtaining written informed consent from each participant, BC patients undergone breast surgery in the period between July 2018 and July 2020 and eligible for adjuvant chemotherapy were recruited. All procedures were in accordance with the ethical standards of the Helsinki Declaration issued in 1975 and later amendments. Exclusion criteria included previous radiation and/or chemotherapy, history of a previous cancer, alterations of breast skin trophism, body mass index (BMI) ≥ 35 kg/m^2^. Moreover, we excluded patients taking other supplements and/or topical treatment/gels. Patients were followed up for a minimum of 6 months and maximum 18 months.

The indication to adjuvant chemotherapy was decided during the Breast Unit multidisciplinary meeting.

We recorded participants’ demographic and anthropometric characteristics (age, weight, height, BMI), serum and metabolic biomarkers, including complete blood count (CBC). Histological diagnosis, tumor staging, and details relating to breast surgery performed were collected.

All patients enrolled in the study underwent chemotherapy cycles including the use of anthracyclines-cyclophosphamide (epirubicin-cyclophosphamide), taxanes (paclitaxel), and/or trastuzumab according to the BC histochemical subtype. The patients were randomly divided into two groups: we administered to one group a combined inositol therapy (topical + oral) (Inositol Group) in addition to chemotherapy and to the other one the chemotherapy in absence of any type of inositol administration (Control Group).

### 2.2. Intervention

BC patients enrolled in the Inositol Group received topical gel containing 5 g of 4% IP6 and capsules containing 390 mg of myo-inositol (kindly granted by Lo.Li. Pharma Srl, Italy). The gel was applied to the entire breast region surgically treated 2 times per day. The capsules were taken by the patients 30 min before each principal meal (2 times per day). Patients began both topical and oral treatment 14 days before starting the adjuvant chemotherapy. In fact, we know from pharmacodynamic and pharmacokinetic studies that IP6 and its intermediate myo-inositol reach the “plateau dose” after 14 days of treatment [25,26]. Moreover, both gel and capsules were taken for the entire duration of the chemotherapy cycles and for 2 weeks after the last cycle.

### 2.3. Blood Sample Collection

Blood samples were collected on fasting condition from a peripheral vein, contralateral to the tumor site, to evaluate the CBC and biochemical parameters. Venous blood sampling was performed at baseline before starting the adjuvant chemotherapy (T0), after the first 2 doses of epirubicin-cyclophosphamide administered once every 3 weeks (T1), at the end of epirubicin-cyclophosphamide cycle (T2), after the first 6 doses of paclitaxel administered once a week (T3) and at the end of the paclitaxel treatment (T4).

### 2.4. Quality-of-Life

For the assessment of health-related quality of life, the European Organization for Research and Treatment of Cancer (EORTC) QLQ-C30 and QLQ-BR23 questionnaires were administered [27,28].

The QLQ-C30 and QLQ-B23 questionnaires were kindly provided to us by the EORTC.

The QLQ-BR23 questionnaire, specific for BC, is divided into different scales, with a score calculated separately for each scale, and evaluates: (i) side effects of systemic therapy, arm and breast pain (symptom scales: the lower the score, the better feels the patient), and (ii) body image, sexual functioning, hair loss and future life prospects (functional scales: the higher the score, the better feels the patient).

The QLQ-C30 questionnaire measures the quality of life in cancer patients through 5 scales that evaluate physical, role-playing, emotional, cognitive, and social functioning (functional scales: the higher the score, the better feels the patient) and quality-of-life related symptoms (fatigue, nausea and vomiting, dyspnea, insomnia, appetite loss, constipation, diarrhea, financial difficulties) (symptom scales: the lower the score, the better feels the patient).

These questionnaires were administered to the patients at different times: before starting the chemotherapy (T0), and at T1, T2, T3 and T4. The scores of the 2 groups were collected and compared with each other.

### 2.5. Statistical Analysis

Descriptive statistics summarizing quantitative variables included median, 25th and 75th percentiles. Wilcoxon-Mann-Whitney test was used to compare quantitative variables between the two treated groups, while Wilcoxon signed rank sum test was performed to compare the changes at each time-point of follow-up from t0 (baseline) for the parameters evaluated in the study in both arms.

Data are presented using box plot. Statistical analysis was implemented at two-sided with a 0.05 significance level, using SAS^®^ version 9.4 (SAS Institute Inc. 100 SAS Campus Drive Cary, NC, USA) and StataTM version 8.2 (StataCorp LLC, College Station, TX, USA).

## 3. Results

### 3.1. Patients’ Characteristics

We initially considered eligible for the study forty BC patients. Four patients did not complete the regimen treatment because transferred to other oncology units. Therefore, a total of 36 BC patients were enrolled, 18 patients randomly assigned to the Inositol Group and 18 patients serving as a Control Group. No dietary supplements neither growth factors have been taken from each group. Anthropometric patients’ characteristics, comorbidities, past medical history information are reported in Table 1. Breast cancer patients had a median age of 52 in the Inositol Group and 58 in the Control Group. An equal number of patients underwent conservative breast surgery or mastectomy and sentinel lymph node biopsy/axillary node dissection (Table 1), and based on BC histochemical subtype, received the same adjuvant chemotherapy regimen documenting a homogeneity between the two groups of patients at baseline, as shown in the Methods Section.

### 3.2. Blood Parameters and Quality of Life between Inositol Group and Control Group at Baseline (T0)

No differences have been documented in terms of CBC values as well as in biochemical parameters (Table 1). Considering the scores obtained from the QLQ-B23 and QLQ-C30 questionnaires administered at baseline, no differences emerged between the two groups, except for the arm symptoms and the quality-of-life related symptoms for which the score was lower in the Inositol Group with respect the Control Group (*p* = 0.029, *p* = 0.001, respectively).

### 3.3. Changes from Baseline in Blood Parameters between Inositol Group and Control Group during the Follow-Up (T1–T4)

**Figure 1:**
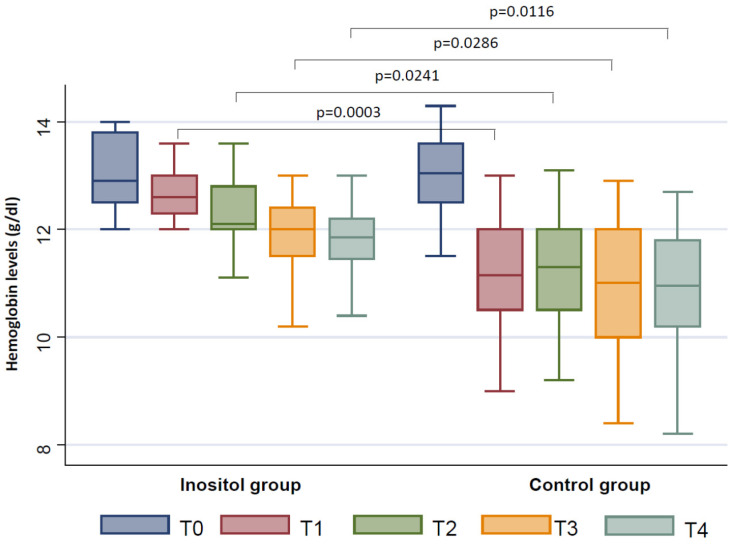
Changes from baseline in hemoglobin levels between the Inositol Group and the Control Group during the follow-up (T1–T4).

At T1, we documented a decrease in the number of erythrocytes in the Inositol Group (*p* = 0.0007) and in the Control Group (*p* < 0.0001), as well as a reduction in hemoglobin levels in the Inositol Group (*p* = 0.0309) and in the Control Group (*p* < 0.0001). Interestingly, the reduction of the hemoglobin levels was lower in the Inositol Group with respect to controls (*p* = 0.0003) (Figure 1). No differences in the number of white blood cells and platelets were documented in both groups.

**Figure 2:**
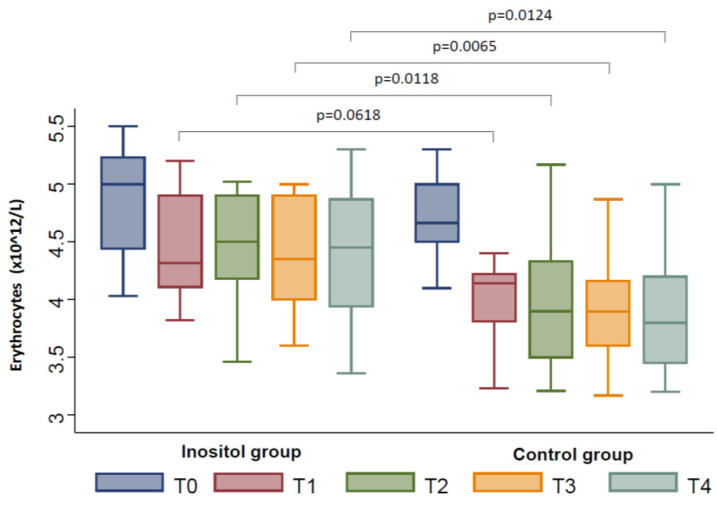
Changes from baseline in erythrocytes between the Inositol Group and the Control Group during the follow-up (T1–T4).

**Figure 3:**
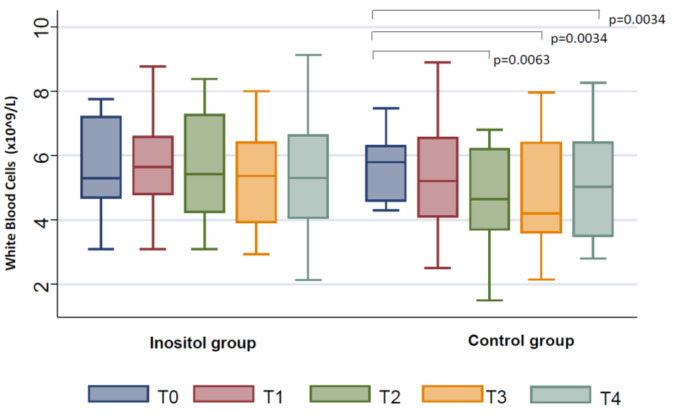
Changes from baseline in white blood cells between the Inositol Group and Control Group during the follow-up (T1–T4).

At T2, we documented an additional decrease in the number of erythrocytes in the Inositol Group (*p* = 0.0026) and in the Control Group (*p* < 0.0001); nevertheless, the decrease was smaller in the Inositol Group with respect to controls (*p* = 0.0118) (Figure 2). Similarly, the hemoglobin levels were lower in the Inositol Group (*p* = 0.0072) and in the Control Group (*p* < 0.0001), although the decrease documented in the Inositol Group was significantly smaller with respect to controls (*p* = 0.0241) (Figure 1). Moreover, we found a significant reduction in the number of the white blood cells in the Control Group (*p* = 0.0063) (Figure 3) but not in the Inositol Group (*p* = 0.844).

At T3, a significant further decrease in the number of erythrocytes was observed in the Inositol Group (*p* = 0.0034), as well as in the Control Group (*p* = 0.0001), although this reduction was lower in the Inositol Group with respect to the Control Group (*p* = 0.0065) (Figure 2). We also observed a lower reduction in hemoglobin levels in the Inositol Group with respect to the Control Group (*p* = 0.0286) (Figure 1). No differences in the number of white blood cells with respect to T0 were documented in the Inositol Group, whereas a significant reduction of this parameter was found in the Control Group (*p* = 0.0034) (Figure 3).

At T4, we confirmed a lower reduction of the hemoglobin levels in the Inositol Group with respect to the Control Group (*p* = 0.0116) (Figure 1) and in terms of red blood cells (*p* = 0.0124) (Figure 2). Moreover, a significant reduction in the number of white blood cells was further confirmed in the Control Group only (*p* = 0.0034) (Figure 3)

### 3.4. Changes from Baseline in EORTC Questionnaire Scores in the Inositol Group and in the Control Group during the Follow-Up (T1-T4)

Considering QLQ-BR23 questionnaire scores, in the Inositol Group we found a significant worsening (decrease) of body image score from baseline to T3 (*p* = 0.0085) and T4 (*p* = 0.0175), systemic therapy side effects score from T0 to T1-T4 (*p* < 0.001) and a significant improvement in breast symptoms score at T4 (*p* = 0.0215) (Table 2), whereas we did not document significant changes from baseline to T4 in QLQ-C30 questionnaire scores in this group (Table 3).

On the other hand, the Control Group showed a significant worsening of the body image score from baseline to T1-T4 (*p* ≤ 0.03) (Table 2) as well as for the score of systemic therapy side effects (*p* ≤ 0.002) (Table 2) and the score of quality-of-life (*p* ≤ 0.001) (Table 3). In particular, regarding the quality-of-life score, we observed a decrease at all the time-points with respect to baseline in terms of all the functioning items included in the score, i.e., physical functioning (*p* ≤ 0.002), role functioning (*p* ≤ 0.01), cognitive functional (*p* ≤ 0.03), and social functioning (*p* ≤ 0.05) (Table 3). In addition, in the same group the symptoms scales of the quality-of-life score resulted lower at all the time-points with respect to baseline for the item fatigue (*p* < 0.001), nausea and vomiting (*p* ≤ 0.03), dyspnea (*p* ≤ 0.01), insomnia (*p* ≤ 0.01) and marginally in appetite loss (*p* = 0.05) (Table 3).

### 3.5. Differences between Inositol Group and Control Group during the Follow-Up (T1–T4) in EORTC Questionnaire Scores

At T1, considering the QLQ-BR23 questionnaire, the breast symptoms score was lower in the Inositol Group with respect to the Control Group (*p* = 0.0296), as well as the arm symptoms score (*p* = 0.0015) and the systemic therapy side effects (*p* = 0.0028), whereas the body image score was higher in the Inositol Group with respect to Control Group (*p* = 0.04) (Table 4). Considering the QLQ-C30 questionnaire, among the quality-of-life related symptoms, only the parameter insomnia presented a significant lower score in the Inositol Group compared to the Control Group (*p* = 0.0442) (Table 5).

At T2, considering the QLQ-BR23 questionnaire, the arm symptoms score was significantly lower in the Inositol Group with respect to controls as well as at T3 and T4 (*p* < 0.01), and no differences in the other scales were detected (Table 4). As for T1, also at T2 the parameter insomnia, among the quality-of-life related symptoms (QLQ-C30 questionnaire), had a significant lower score in the Inositol Group with respect to Control Group (*p* = 0.018), whereas the physical functioning item presented a higher score in the Inositol Group compared to the Control Group (*p* = 0.04) (Table 5).

At T3, no differences in the scores obtained from the QLQ-BR23 scales were detected (Table 4). Interestingly, for the questionnaire QLQ-C30 we documented a significant improvement in the quality-of-life score in the Inositol Group with respect to controls (*p* = 0.0243) specifically for the physical functioning item (*p* = 0.0017) and the insomnia item (*p* = 0.042) (Table 5).

At T4, for the functional scales of QLQ-C30 questionnaire, we documented an overall improvement of quality-of-life score (*p* = 0.0431) and a higher score for the physical functioning item (*p* = 0.0003) in the Inositol Group with respect to controls (Table 5), whereas, a lower score for the fatigue item in the Inositol Group with respect to controls (*p* = 0.0092) as well as for nausea and vomiting (*p* = 0.0020), the dyspnea (*p* = 0.0530), the insomnia (*p* = 0.0043), and the appetite loss (*p* = 0.0147) (Table 5).

## 4. Discussion

This is the first study evaluating the combined administration of inositol (oral + topical) in BC patients treated with upfront surgery and adjuvant chemotherapy. Previous clinical studies investigated the use of IP6 in BC patients documenting the role of inositol in reducing systemic complications and side effects of adjuvant chemotherapy, including hematopoietic cytotoxicity, with an improvement in patients’ quality of life [21,22]. However, there were no studies investigating the role of inositol on local symptoms in the post-operative course of breast surgery, in particular on the post-operative symptoms/complications affecting the breast and the upper limb in BC patients, i.e., possible seroma, hematoma or surgical site infection in the immediate postoperative period, as well as sequelae that can occur more or less later, such as lymphedema of the upper limb resulting in functional limitations.

Interestingly, an experimental study found that inositol is absorbed through the skin using either gel or cream formulation; however, urinary IP6 values resulted higher when using the gel in combination with oral IP6 compared to oral IP6 treatment alone, due to the formation of insoluble species in the gastrointestinal tract highly limiting its intestinal absorption [25].

In our study, the patients in the Inositol Group and those in the Control Group did not show significant differences at baseline in terms of anthropometric and metabolic parameters, neither in terms of surgical procedures performed and in the post-operative course. Moreover, the two groups were comparable in terms of stage of BC, involvement of the axillary lymph nodes and surgical procedures performed.

Therefore, the differences observed during adjuvant therapy in terms of post-operative symptoms cannot be due to an initial imbalance between the two groups resulting from different surgical procedures.

During the adjuvant chemotherapy treatment, the erythrocytes showed in both groups a significant tendency to decrease. However, a less pronounced reduction was detected in patients of the Inositol Group, who maintained relatively higher and stable red blood cell values in all the time-points analyzed. The stability in the hemoglobin levels confirmed the lower impact of chemotherapy on the red series in the patients treated with inositol, reinforcing the hypothesis that inositol may have had a protective role on the erythroid line. The study by Lamarre et al. described how IP6 was able to limit, in hypoxic conditions, the sickling process of red blood cells in patients with sickle cell anemia, which is a role already confirmed in vitro by Bourgeaux et al. [14,29]. In particular, when transfusing red blood cells obtained from the incorporation of IP6 into patients with sickle cell anemia, a reduced tendency to deformability and sickling of red blood cells was documented, as well as a reduced tendency to aggregation and blood viscosity [14]. This shows an important erythrocyte stabilization effect mediated by inositol, especially in hypoxic conditions. This is determined by the fact that IP6 is a powerful allosteric effector of the hemoglobin and guarantees the possibility that red blood cells may release more oxygen to peripheral tissues, limiting hypoxia [14]. Moreover, the IP6 has direct effects on the hematopoietic process, acting on red blood cells maintaining their integrity through its calcium chelating action and improving their affinity with oxygen [14]. Inositol appears to have opposite effects on normal cells and cancer cells. Unlike what it does in cancer cells, IP6 activates PI3K in healthy cells, improving their metabolism, as well as Akt pathway, improving cell survival [22,30]. However, the role of inositol in relation to the erythrocyte line is still only partially understood and deserves further studies.

According with other authors [14], we also documented that in the Inositol Group the number of white blood cells remained relatively stable during the adjuvant treatment compared to the Control Group, which presented a significant reduction of these cells from T0 to T4.

The majority of the patients undergoing chemotherapy have some abnormalities in their complete blood count, primarily in the number of leukocytes and platelets. Changes in the complete blood count, in particular low white blood cells, induce the oncologists to postpone the chemotherapy cycles in order to prevent any additional undesirable side effects, delaying the completion of anti-cancer treatments. For this reason, the maintenance of normal blood cells values during therapies is an important goal to guarantee the appropriate chemotherapy cycles schedule [31,32].

In our cohort, the data collected from the QLQ-BR23 questionnaires regarding symptoms of the breast related to the surgical-site did not document significant differences between the two groups at baseline or during the chemotherapy treatments, except at T1, which was the time-point corresponding to the first chemotherapy cycle. However, if we analyze the trend of breast symptoms in each group, the scores appear ameliorated (lower) in the treatment group with respect to controls during the treatment, and this was particularly evident at the end of chemotherapy cycles (considering that higher scores indicate worse symptoms).

Interestingly, considering the arm symptoms, which are the most disabling during the post-operative period, mainly due to the surgical damage and the possible fibrosis development, we documented at each time of the study (from T1 to T4) a significant improvement in the treatment group with respect to controls.

No previous studies documented the impact of IP6 or myo-inositol on local symptoms in the post-operative course in of BC patients. Multiple effects, i.e., antioxidant, anti-inflammatory and anti-fibrinogenetic of inositol are known, including its ability to regulate molecular pathways involved in the inflammatory cascade, such as NF-kB, PGE2 and COX−2 [13,33,34], as well as the ability to reduce fibrosis through the regulation of TGF-beta [13,33]. A randomized study investigated the impact of a combination therapy, based on myo-inositol, betaine and boswellia, on mastalgia and on breast symptoms reported by patients with benign breast lesions, mainly due to increased breast density [35]. The improvement of mastalgia was significantly higher in the treated group with respect to the Control Group, documenting a potential role of inositol in reducing “breast density” and breast pain. Moreover, the data obtained in a study investigating the effects of an oral supplementation of an association of boswellia, betaine and myo-inositol in the treatment of mammographic breast density showed that women with high breast density experienced a significant clinical benefit when treated with the supplement [36]. The authors hypothesized that myo-inositol may improve the clinical features of breast density by interfering with tissue metabolism at local and systemic level [36]. In fact, myo-inositol modulates metabolic and hormonal pattern, by improving glucose uptake and insulin sensitivity and normalizing lipid metabolism [37]. In addition, myo-inositol has shown to prevent pulmonary fibrosis after inflammatory injury [38], contrasting inflammation-induced fibrosis by modulating TGF-beta activity [39]. TGF-beta is a potent pro-fibrogenic agent inducing collagen synthesis and regulating the balance between matrix-degrading metalloproteinases and their inhibitors [39]. In this light, TGF-beta down-regulation, modulated by myo-inositol, may improve the local density and edema developed in inflammatory conditions or during microbe-induced infection/inflammation, which may be a complication of the surgical procedure.

The symptom scales evaluating the quality of life through the QLQ-C30 questionnaires utilized during the study presented a significant worsen course at all the time-points only in the Control Group and no modifications were documented in the intervention group. Interestingly, the symptom fatigue had a significant increase in the Control Group at all the time-points, likely due to the decrease of the CBC parameters documented in control BC patients. A similar trend was documented for the symptoms insomnia and body image, which may be in part explained by the fact that low levels of myoinositol have been linked to the pathophysiology of depression and concomitant sleep symptoms [40,41].

The Control Group presented also a significant appetite loss, not documented in the intervention group, most probably because of the anti-inflammatory and anti-oxidant properties of inositol, considering that in cancer often the neuroinflammation underlies the loss of appetite [42]. The clinical relevance of the loss of appetite (anorexia) and low food intake in cancer patients is represented also by the consequent body weight loss which relates with poor outcomes before and during anticancer treatments [43].

In this perspective, our data provide encouraging results considering that in cancer patients, including BC patients, quality-of-life and adverse effects represent a very critical aspect during cancer journey and new therapeutic integrated advances may improve patient’s outcome(s) in different stages of the tumor [44].

We acknowledge the limitations of our study. First, the study was conducted on a small number of patients and we did not provide the Control Group with gel and tabs containing a placebo, although our results confirmed previous observations and documented that myo-inositol and IP6, when included in adjuvant chemotherapy regimen for BC, significantly improved patients’ quality of life and protected patients from important reduction in the number of erythrocytes and leukocytes.

Moreover, we did not focus on objective assessment of local breast and arm post-surgical conditions, investigating only subjective symptoms assessment.

Even though the results of our study, conducted on a small cohort, are encouraging, further multicentric clinical studies on a larger number of patients are necessary for a more detailed evaluation of the impact that oral myo-inositol + topical IP6 application may have on BC patients outcomes.

## Figures and Tables

**Table 1:** Patients’ characteristics at baseline.

All Participants *N* = 36	Inositol Group *N* = 18	Control Group *N* = 18
Age, years	52.0 (48.0; 62.0)	58.0 (53.0; 69.0)
Body weight, kg	65.0 (63.0; 77.0)	64.00 (62.0; 70.0)
BMI, weight (kg)/height^2^ (m)	25.1 (23.4; 27.9)	25.3 (23.9; 26.8)
RBC, ×10^12^/L	5.0 (4.44; 5.23)	4.7 (4.54; 5.0)
Hb levels, g/dL	12.9 (12.5; 13.8)	13.05 (12.5; 13.6)
WBC, ×10^9^/L	5.3 (4.7; 7.2)	5.8 (4.6; 6.3)
Comorbidities:		
*Diabetes mellitus (y/no)*	0/18	0/18
*Arterial hypertensione (y/no)*	4/14	4/14
*Hyperlipidemia (y/no)*	2/16	3/15
Type of breast surgery		
*Breast conserving surgery/*		
*Mastectomy, n*	6/12	7/11
Type of axillary surgery		
*SLN biopsy/ALND, n*	10/8	11/7
Tumor diameter, cm	2.0 (1.2; 2.24)	1.6 (1.23; 2.5)
Removed axillary lymph nodes, n	5.0 (3.0; 6.0)	4.0 (2.0; 11.0)

Data are shown as median (IQR). *p* values are not significant for the patient’ characteristics shown between groups. Abbreviations include: BMI, body mass index; RBC, red blood cell; Hb, hemoglobin; WBC, white blood cell; SLN, sentinel lymph node; ALND, axillary lymph node dissection.

**Table 2:** Variations of QLQ-BR23 questionnaires’ scores from baseline (T0) to T1-T4 in the Inositol Group and in the Control Group.

All Participants *N* = 36		Inositol Group *N* = 18	*p* Value	Control Group *N* = 18	*p* Value
***QLQ-BR23 Functional scales***					
Body image	T0 (baseline)	83.3 (66.7; 91.7)		79.2 (66.7; 91.7)	
	T1–T0	0.0 (−8.3; 8.3)	>0.99	−8.3 (−25.0; 0.0)	**0.0007**
	T2–T0	−8.3 (−25.0; 0.0)	0.1484	−8.3 (−33.3; 0.0)	**0.001**
	T3–T0	−16.7 (−33.3; 0.0)	**0.0085**	−8.3 (−58.3; 0.0)	**0.0065**
	T4–T0	−16.7 (−33.3; 0.0)	**0.0175**	−8.3 (−58.3; 0.0)	**0.0044**
***QLQ-BR23 Symptom scales***					
Systemic therapy side effects T0		4.8 (0.0; 14.3)		4.7 (0.0; 14.3)	
	T1–T0	19.1 (9.5; 3.8)	**0.001**	38.1 (19.1; 52.4)	**<0.0001**
	T2–T0	14.3 (4.8; 28.6)	**0.0002**	33.3 (16.7; 52.4)	**<0.0001**
	T3–T0	19.1 (7.1; 40.5)	**0.0002**	42.9 (23.8; 52.4)	**<0.0001**
	T4–T0	28.6 (14.3; 57.1)	**0.0001**	52.4 (30.9; 69.1)	**<0.0001**
Breast symptoms	T0 (baseline)	16.7 (8.3; 25.0)		8.3 (0.0; 41.7)	
	T1–T0	0.0 (−16.7; 0.0)	0.1465	0.0 (0.0; 8.3)	0.418
	T2–T0	−4.2 (−16.7; 0.0)	0.1216	0.0 (−8.3; 8.3)	0.4906
	T3–T0	0.0 (−16.7; 0.0)	0.0569	0.0 (0.0; 8.3)	0.8218
	T4–T0	−8.3 (−16.7; 0.0)	**0.0215**	0.0 (−8.3; 16.7)	0.9971
Arm symptoms	T0 (baseline)	16.7 (0.0; 22.2)		22.2 (11.1; 33.3)	
	T1–T0	0.0 (−11.1; 0.0)	0.2949	0.0 (0.0; 22.2)	**0.0195**
	T2–T0	0.0 (−11.1; 0.0)	0.4023	5.6 (−11.1; 22.2)	0.0947
	T3–T0	0.0 (−11.1; 22.2)	0.5778	11.1 (0.0; 33.3)	**0.0221**
	T4–T0	0.0 (−22.2; 0.0)	>0.99	11.1 (11.1; 33.3)	**<0.0001**

**Table 3:** Variations of QLQ-C30 questionnaires’ scores from baseline (T0) to T1-T4 in the Inositol Group and in the Control Group.

All Participants *N* = 36			Inositol Group *N* = 18	*p* Value	Control Group *N* = 18	*p* Value
***QLQ-C30 Functional scales***						
Quality of life	T0 (baseline)		54.2 (33.3; 58.3)		66.7 (66.7; 83.3)	
	T1–T0		0.0 (0.0; 16.7)	0.2168	−33.3 (−41.7; −8,33)	**0.0001**
	T2–T0		0.0 (−8.3; 16.7)	0.5757	−25.0 (−33.3; 0.0)	**0.0007**
	T3–T0		0.0 (−16.7; 8.3)	0.6797	−33.3 (−50.0; −16.7)	**0.0001**
	T4–T0		0.0 (−16.7; 16.7)	0.9314	−25.0 (−50.0; −16.7)	**<0.0001**
Physical functioning	T0 (baseline)		86.7 (73.3; 86.7)		86.7 (80.0; 100.0)	
	T1–T0		−3.3 (−20.0; 6.7)	0.2507	−26.7 (−40.0; −6.7)	**<0.0001**
	T2–T0		0.0 (−13.3; 20.0)	0.7024	−26.7 (−46.7; −6.7)	**0.0002**
	T3–T0		−10.0 (−26.7; 13.3)	0.3068	−43.3 (−46.7; −13.3)	**<0.0001**
	T4–T0		−10.0 (−26.7; 13.3)	0.106	−46.7 (−66.7; −26.7)	**<0.0001**
Role functioning	T0 (baseline)		66.7 (50.0; 83.3)		75.0 (66.7; 100.0)	
	T1–T0		0.0 (−16.7; 33.3)	0.9204	−16.7 (−33.3; 0.0)	**0.001**
	T2–T0		0.0 (−16.7; 33.3)	0.7852	−16.7 (−33.3; 16.7)	**0.0347**
	T3–T0		−16.7 (−33.3; 16.7)	0.3326	−33.3 (−50.0; 0.0)	**0.0033**
	T4–T0		−16.7 (−33.3; 0.0)	0.2574	−33.3 (−66.7; −16.7)	**0.0003**
Cognitive functioning	T0 (baseline)		83.3 (50.0; 100.0)		100.0 (83.3; 100.0)	
	T1–T0		0.0 (−16.7; 16.7)	0.9258	−16.7 (−66.7; 0.0)	**0.002**
	T2–T0		0.0 (−16.7; 0.0)	0.4307	0.0 (−50.0; 0.0)	**0.0195**
	T3–T0		−8.3 (−33.3; 16.7)	0.1913	−25.0 (−50.0; 0.0)	**0.0029**
	T4–T0		0.0 (−33.3; 16.7)	0.2955	−16.7 (−50.0; 0.0)	**0.001**
Social functioning	T0 (baseline)		75.0 (66.7; 100.0)		83.3 (66.7; 100.0)	
	T1–T0		−8.3 (−16.7; 0.0)	0.0508	−16.7 (−33.3; 0.0)	**0.0005**
	T2–T0		−8.3 (−33.3; 0.0)	0.1011	0.0 (−16.7; 0.0)	**0.0444**
	T3–T0		−16.7 (−50.0; 0.0)	0.1653	−25.0 (−50.0; 0.0)	**0.0034**
	T4–T0		−16.7 (−50.0; 0.0)	0.139	−33.3 (−50.0; 0.0)	**0.0017**
***QLQ-C30 Symptom scales***						
Fatigue	T0 (baseline)		33.3 (11.1; 44.4)		16.7 (0.0; 33.3)	
	T1–T0		0.0 (−11.1; 33.3)	0.2867	44.4 (0.0; 55.6)	**0.0002**
	T2–T0		0.0 (−11.1; 33.3)	0.2771	38.9 (0.0; 55.6)	**0.0007**
	T3–T0		22.2 (−11.1; 33,.3)	0.1015	44.4 (22.2; 66.7)	**<0.0001**
	T4–T0		16.7 (0.0; 33.3)	0.0854	66.7 (22.2; 77.8)	**<0.0001**
Nausea and vomiting	T0 (baseline)		0.0 (0.0; 16.7)		0.0 (0.0; 0.0)	
	T1–T0		0.0 (0.0; 33.3)	0.2227	16.7 (0.0; 50.0)	**0.0059**
	T2–T0		0.0 (0.0; 16.7)	0.7070	8.3 (0.0; 33.3)	**0.0059**
	T3–T0		0.0 (0.0; 16.7)	0.9609	8.3 (0.0; 50.0)	**0.0322**
	T4–T0		0.0 (−16.7; 0.0)	0.6230	25.0 (0.0; 66.7)	**0.0022**
Dispnoea	T0 (baseline)		0.0 (0.0; 33.3)		0.0 (0.0; 33.3)	
	T1–T0		0.0 (0.0; 33.3)	0.0625	0.0 (0.0; 33.3)	**0.0078**
	T2–T0		0.0 (0.0; 33.3)	0.0625	16.7 (0.0; 33.3)	**0.0137**
	T3–T0		0.0 (0.0; 33.3)	**0.0156**	33.3 (0.0; 33.3)	**0.002**
	T4–T0		16.7 (0.0; 33.3)	**0.0215**	33.3 (0.0; 33.3)	**0.0013**
Insomnia	T0 (baseline)		0.0 (0.0; 66.7)		33.3 (0.0; 33.3)	
	T1–T0		0.0 (0.0; 0.0)	0.5703	16.7 (0.0; 66.7)	**0.0117**
	T2–T0		0.0 (−33.3; 33.3)	0.5820	16.7 (0.0; 66.7)	**0.0059**
	T3–T0		0.0 (−33.3; 33.3)	0.8621	0.0 (0.0; 66.7)	**0.0078**
	T4–T0		0.0 (−33.3; 33.3)	0.8267	33.3 (0.0; 66.7)	**0.0007**
Appetite loss	T0 (baseline)		0.0 (0.0; 0.0)		0.0 (0.0; 0.0)	
	T1–T0		0.0 (0.0; 33.3)	0.6719	33.3 (0.0; 33.3)	**0.0471**
	T2–T0		0.0 (0.0; 33.3)	0.8281	0.0 (0.0; 33.3)	0.2734
	T3–T0		0.0 (0.0; 0.0)	0.8438	16.7 (0.0; 66.7)	**0.0156**
	T4–T0		0.0 (0.0; 0.0)	0.8828	50.0 (0.0; 66.7)	**0.0037**

**Table 4:** Differences between the Inositol Group and the Control Group during the follow-up (T1-T4) in QLQ-BR23 questionnaire scores.

All Participants *N* = 36		Inositol Group *N* = 18	Control Group *N* = 18	*p* Value
***QLQ-BR23 Functional scales***				
Body image				
	T1–T0	83.3 (66.7; 83.3)	66.7 (33.3; 83.3)	**0.0422**
	T2–T0	66.7 (66.7; 83.3)	66.7 (33.3; 66.7)	0.1461
	T3–T0	66.7 (66.7; 75.0)	58.3 (8.3; 100.0)	0.5191
	T4–T0	66.7 (58.3; 66.7)	62.5 (8.3; 75.0)	0.4971
***QLQ-BR23 Symptom scales***				
Systemic therapy side effects				
	T1–T0	23.8 (19.1; 33.3)	38.1 (33.3; 57.1)	**0.0028**
	T2–T0	28.6 (14.3; 38.1)	40.5 (21.4; 61.9)	0.0823
	T3–T0	33.3 (19.1; 42.9)	47.6 (28.6; 66.7)	**0.0429**
	T4–T0	38.1 (19.1; 57.1)	57.1 (40.5; 71.4)	**0.0193**
Breast symptoms				
	T1–T0	8.3 (0.0; 16.7)	16.7 (8.3; 33.3)	**0.0296**
	T2–T0	8.3 (0.0; 16.7)	12.5 (0.0; 33.3)	0.1788
	T3–T0	8.3 (0.0; 16.7)	16.7 (8.3; 33.3)	0.1003
	T4–T0	4.2 (0.0; 16.7)	16.7 (8.3; 33.3)	**0.0396**
Arm symptoms				
	T1–T0	0.0 (0.0; 22.2)	27.8 (22.2; 55.6)	**0.0015**
	T2–T0	0.0 (0.0; 22.2)	22.2 (11.1; 55.6)	**0.0053**
	T3–T0	11.1 (0.0; 22.2)	33.3 (11.1; 66.7)	**0.0079**
	T4–T0	0.0 (0.0; 22.2)	50.0 (33.3; 66.7)	**<0.0001**

**Table 5:** Differences between the Inositol Group and the Control Group during the follow-up (T1-T4) in QLQ-C30 questionnaire scores.

All Participants *N* = 36		Inositol Group *N* = 18	Control Group *N* = 18	*p* Value
***QLQ-C30 Functional scales***				
Quality of life				
	T1–T0	50.0 (41.7; 66.7)	37.5 (33.3; 50.0)	0.052
	T2–T0	50.0 (50.0; 58.3)	50.0 (33.3; 66.7)	0.6291
	T3–T0	54.2 (41.7; 58.3)	37.5 (16.7; 50.0)	**0.0243**
	T4–T0	58.3 (33.3; 66.7)	33.3 (16.7; 58.3)	**0.0431**
Physical functioning				
	T1–T0	76.7 (66.7; 86.7)	56.7 (40.0; 80.0)	0.1012
	T2–T0	73.3 (66.7; 86.7)	40.0 (40.0; 86.7)	**0.0409**
	T3–T0	73.3 (66.7; 86.7)	40.0 (33.3; 66.7)	**0.0017**
	T4–T0	73.3 (46.7; 80.0)	33.3 (33.3; 53.3)	**0.0003**
Role functioning				
	T1–T0	66.7 (33.3; 83.3)	50.0 (33.3; 66.7)	0.386
	T2–T0	66.7 (50.0; 100.0)	50.0 (50.0; 83.3)	0.5828
	T3–T0	50.0 (33.3; 66.7)	50.0 (33.3; 83.3)	0.6873
	T4–T0	33.3 (33.3; 83.3)	33.3 (33.3; 50.0)	0.2841
Cognitive functioning				
	T1–T0	83.3 (50.0; 100.0)	66.7 (33.3; 83.3)	0.1538
	T2–T0	75.0 (50.0; 83.3)	83.3 (33.3; 100.0)	0.6604
	T3–T0	66.7 (33.3; 100.0)	66.7 (33.3; 83.3)	0.6999
	T4–T0	66.7 (50.0; 100.0)	66.7 (33.3; 83.3)	0.2949
Social functioning				
	T1–T0	66.7 (50.0; 83.3)	66.7 (50.0; 66.7)	0.5794
	T2–T0	58.3 (50.0; 83.3)	66.7 (66.7; 100.0)	0.1503
	T3–T0	50.0 (33.3; 83.3)	66.7 (33.3; 66.7)	0.8187
	T4–T0	58.3 (33.3; 83.3)	33.3 (33.3; 66.7)	0.5849
***QLQ-C30 Symptom scales***				
Fatigue				
	T1–T0	33.3 (33.3; 55.6)	55.6 (44.4; 66.7)	0.077
	T2–T0	44.4 (33.3; 66.7)	44.4 (33.3; 55.6)	0.0714
	T3–T0	50.0 (33.3; 66.7)	61.1 (44.4; 88.9)	0.0883
	T4–T0	55.6 (33.3; 66.7)	72.2 (66.7; 88.9)	**0.0092**
Nausea and vomiting				
	T1–T0	16.7 (0.0; 33.3)	16.7 (0.0; 50.0)	0.2878
	T2–T0	16.7 (0.0; 16.7)	16.7 (0.0; 50.0)	0.3178
	T3–T0	8.3 (0.0; 16.7)	16.7 (0.0; 50.0)	0.1073
	T4–T0	0.0 (0.0; 16.7)	33.3 (0.0; 66.7)	**0.002**
Dispnoea				
	T1–T0	33.3 (0.0; 33.3)	33.3 (0.0; 66.7)	0.2709
	T2–T0	33.3 (0.0; 33.3)	33.3 (0.0; 66.7)	0.3438
	T3–T0	33.3 (0.0; 66.7)	33.3 (33.3; 66.7)	0.2529
	T4–T0	33.3 (0.0; 33.3)	33.3 (33.3; 66.7)	**0.043**
Insomnia				
	T1–T0	33.3 (0.0; 33.3)	50.0 (33.3; 66.7)	**0.0442**
	T2–T0	33.3 (0.0; 33.3)	66.7 (33.3; 66.7)	**0.0182**
	T3–T0	33.3 (0.0; 33.3)	66.7 (33.3; 66.7)	**0.0424**
	T4–T0	33.3 (33.3; 33.3)	66.7 (33.3; 66.7)	**0.0043**
Appetite loss				
	T1–T0	0.0 (0.0; 33.3)	33.3 (0.0; 33.3)	0.3689
	T2–T0	0.0 (0.0; 33.3)	0.0 (0.0; 33.3)	0.8373
	T3–T0	0.0 (0.0; 33.3)	33.3 (0.0; 66.7)	0.1008
	T4–T0	0.0 (0.0; 0.0)	50.0 (0.0; 66.7)	**0.0147**

## Data Availability

Research data are available at the Corresponding author upon request.
